# The screening of visual impairment among preschool children in an urban population in Malaysia; the Kuching pediatric eye study: a cross sectional study

**DOI:** 10.1186/1471-2415-13-16

**Published:** 2013-04-19

**Authors:** Mallika Premsenthil, Rose Manju, Asokumaran Thanaraj, Syed Alwi Syed Abdul Rahman, Tan Aik Kah

**Affiliations:** 1 Department of Ophthalmology, Faculty of Medicine and Health Sciences, Universiti Malaysia Sarawak (UNIMAS), Lot 77, Seksyen 22, Kuching Town Land District, Jalan Tun Ahmad Zaidi Adruce, Kuching, Sarawak, 93150, Malaysia; 2 Department of Nursing, Faculty of Medicine and Health sciences, University Malaysia Sarawak (UNIMAS), Lot 77, Seksyen 22, Kuching Town Land District, Jalan Tun Ahmad Zaidi Adruce, Kuching, Sarawak, 93150, Malaysia; 3 Department of Ophthalmology, Sarawak General Hospital, Kuching, Sarawak, 93150, Malaysia; 4 Department of Family Medicine, Faculty of Medicine and Health Sciences, Universiti Malaysia Sarawak (UNIMAS), Lot 77, Seksyen 22, Kuching Town Land District, Jalan Tun Ahmad Zaidi Adruce, Kuching, Sarawak, 93150, Malaysia

**Keywords:** Preschool children, Eye screening, Prevalence, Visual impairment, Distant visual acuity, Stereopsis, Amblyopia

## Abstract

**Background:**

To screen for visual impairment in Malaysian preschool children.

**Methods:**

Visual screening was conducted in 400 preschool children aged 4 to 6 years. The screening involved two basic procedures; the distant visual acuity test using the Sheridan Gardiner chart and the depth perception test using the Langs stereoacuity test. Criteria for referral were a visual acuity of 6/12 or less in the better eye or a fail in the depth perception test.

**Results:**

The prevalence of visual impairment was 5% (95% confidence interval [CI] = 3.3, 7.6). Of the 400 preschool children screened, 20 of them failed the distant visual acuity test or the stereopsis test. Refractive errors were the most common cause of visual impairment (95%, 95% CI = 76.2, 98.8); myopic astigmatism was the commonest type of refractive error (63.2%, 95% CI = 40.8, 80.9).

**Conclusion:**

The study is a small but important step in the effort to understand the problem of visual impairment among our preschool children. Our study showed that it is feasible to measure distant visual acuity and stereopsis in this age group.

## Background

Vision is an important requirement for learning and plays a critical role in the development of a child during the first three years of life. Children use their sight to strengthen motor functions, establish parent–child bonding, build picture perception and gain their balance [1]. Children may enter school with vision problems. Sub-optimal vision could lead to poor school performance, lack of interest in schooling, and dropping out of school. Sometimes an underlying vision problem can manifest as behavioral problems like learning disabilities, dyslexia and attention deficit disorder [2]. The common eye problems that can occur in children of preschool and early school age include amblyopia, strabismus and refractive errors [3]. Early detection provides the best opportunity for effective treatment. The American Academy of Pediatrics recommends early vision screening at 3 years of age [4].

Amblyopia affects 5% of the preschool children and is potentially treatable [5]. The two common causes of amblyopia are strabismus and refractive errors. Early detection is critical because it increases the likelihood of successful treatment. Preschool screening programs may result in better visual outcome than screening at school entry. In Malaysia, the nationwide eye screening program effectively covers only primary school children who are 7 years of age and above, but does not include children in the preschool age (6 years and below). To date, there is no data on the prevalence of vision problems among preschool children in Borneo Island. In this report, we provide information on the magnitude and type of visual problems. This data will aid in the estimation of the need for an eye screening program among preschool children in our population.

## Methods

The Kuching Pediatric Eye Study research protocol was approved by the University Malaysia Sarawak (UNIMAS) Human Subjects Ethics committee [Project ID: SGS/01(S63)/761/2010(38)]. The study was conducted in accordance with the Helsinki’s declaration and written informed consent obtained from parents of all participants.

### Design and sample

The Kuching Pediatric Eye Study was a cross-sectional study involving 400 preschool children, aged 4 to 6 years in Kuching district, Malaysia. Data was collected from the year 2010 to 2011. Kuching has a population of 617,887. About 8.34% (51,530) are preschool children [6]. The list of kindergartens in Kuching district was obtained from the Sarawak State Education Council. The number of kindergarten in Kuching district included 122 government and 89 private preschools. Preschool children were selected by simple random sampling. Numbers were randomly assigned to all the preschools and the screening population was selected by SPSS random selection methods. Sample size was calculated for an expected prevalence rate of 20.6% and for a precision of 4.0%.

### Site inspection and informed consent

After identification of the kindergartens, the respective principals were approached and informed about the study. Upon agreement, the schools were inspected for the suitability of the screening process. A school is considered suitable if there is a room with more than 4 meter long which is free from distractions. The light level should be adequate (at least 300 lux in the room and test chart illumination of about 500 lux). Consent forms, questionnaires and information sheets were distributed to the parents two weeks before the screening procedure. The consent forms, questionnaires and information sheets were written in three major languages (English, Chinese and Malay). The questionnaire had information on demographics (gender, age and ethnicity), family eye history, preterm history, medical history and history of any ocular complaints. On the day of screening the consent forms and the questionnaires were collected. Children whose parents gave their written consent to participate in this study were included for the eye screening.

### Vision screening

All preschool children underwent two basic screening procedures which included distant visual acuity test and depth perception test. Measurement of the distant visual acuity was performed using the Sheridan Gardiner Test Complete (Keeler, UK) and the Cardiff charts. Depth perception test or stereopsis assessment was performed using the Langs stereotest. These tests were performed by optometrists and trained staff nurses. The children were subjected to distant visual acuity test first followed by stereopsis assessment. Every child underwent a pretest before the actual assessment of the test procedure. The distant visual acuity test was measured monocularly at a distance of 6 meter. The right eye was tested first before the left eye. The test results of both the eyes were recorded separately. Stereopsis was measured binocularly and the results were recorded as “pass” if the child can identify the figures in the chart correctly and fail if the child was unable to locate the pictures. Children whose visual acuity was worse than 6/12 in one or both the eyes and those who fail in the depth perception test were referred to the eye clinic for a detailed eye examination [7].

### Referral and further evaluation

Children who needed eye referral were sent home with a letter to inform their parents about their condition and also a referral letter to the eye clinic for a comprehensive eye examination. Examinations in the eye clinic were performed by trained ophthalmologists. These include visual acuity assessment, cover-uncover and alternate cover tests, ocular movements, cycloplegic refraction, slit-lamp examination and dilated fundus examination. Significant refractive error were defined as hyperopia ≥3.00 diopters (D), myopia ≥1.00 D or astigmatism ≥1.50 D in either eye, or anisometropia ≥ 2.00 D [8]. Children with underlying refractive errors were prescribed glasses and children with other ocular problems were managed accordingly. Amblyopia was defined as visual acuity worse than 0.3 LogMAR (worse than 20/40) in the affected eye and/or a 2 LogMAR line difference between the two eyes and the presence of an amblyogenic factor [9].

### Statistical analysis

Data entry, cleaning and analyses were performed using SPSS 17.0 for windows. Data analysis was carried out by PS Mallika. Descriptive statistical analysis was performed and results were reported. Categorical data analysis was performed by using either chi-square test or Fishers exact test as appropriate.

## Results

### Demography

All the schools inspected were able to provide a suitable environment for vision screening. Of the 450 preschool children who were eligible to participate in the study, 400 (88.8%) were screened for eye diseases, 30 (6.6%) were absent, and 20 (4.4%) refused to participate. The demographic profile of our study population is given in Table 1. Male: female ratio was almost 1:1. Majority of the children were in the 6 years old age group. Mean age was 5.33 ± 0.69 years. Ethnic composition in the different age groups is given in Table 2. About 80% of the children had visual acuity of 6/6 in both the eyes (Table 3).

**Table 1:** Demographic data

**Variables**	***n***	**%**
Gender		
Male	196	49
Female	204	51
Age Group (Years)		
4	51	12.8
5	163	40.8
6	186	46.5
Race		
Malay	115	28.8
Chinese	131	32.8
Iban	60	15
Bidayuh	76	19
Others	18	4.5

**Table 2:** Ethnic composition in different age groups

	**Ethnicity**										**Total**	
**Age (years)**	**Malay**		**Chinese**		**Iban**		**Bidayuh**		**Others**			
	***n***	**%**	***n***	**%**	***n***	**%**	***n***	**%**	***n***	**%**	***n***	**%**
4	**4**	1.0	**33**	8.25	**8**	2.0	**3**	0.75	**3**	0.75	**51**	12.75
5	**49**	12.25	**44**	11.0	**30**	7.5	**36**	9.0	**4**	1.0	**163**	40.75
6	**62**	15.5	**54**	13.5	**22**	5.5	**37**	9.25	**11**	2.75	**186**	46.5
Total	**115**	28.75	**131**	32.75	**60**	15.0	**76**	19.0	**18**	4.5	**400**	100.0

**Table 3:** Distribution of visual acuity in both eyes

	**Right eye**		**Left eye**	
**Visual acuity**	***n***	**%**	***n***	**%**
6⁄6	335	83.75	323	80.75
6⁄9	48	12.00	60	15.00
6⁄12	12	3.00	10	2.50
6⁄18	3	0.75	5	1.25
6⁄24	1	0.25	1	0.25
6⁄36	0	0.00	0	0.00
6⁄60	1	0.25	0	0.00
< 6⁄60	0	0.00	1	0.25
Total	400	100	400	100

The prevalence of visual impairment in our population was 5% (95% CI = 3.3%, 7.6%). Ninety six percent of the children passed the distant vision screening test and 99% of them passed the stereopsis test. Of the 20 who did not pass the distant visual acuity test, one was found to have speech disorder; the others were due to eye problems. Visual impairment was common among boys (*n* = 13, 65%) compared to girls (*n* = 7, 35%). However, the difference is not statistically significant (p = 0.142). Children in age group of 6 were found to have visual impairment more compared to other age groups (statistically not significant, p = 0.732) (Table 4). Children from the Bidayuh ethnic group had higher number of visual impairment (*n* = 8) compared other ethnic groups (statistically not significant, p = 0.171). A positive family history of eye disease was present in 24% of children and the most common was family history of refractive errors. History of medical illness was seen in 6% of children and prematurity in 6.8%. Twenty eight percent of the children had ocular complaints and the commonest ocular complaint was photophobia.

**Table 4:** Distribution of visual impairment and age group

		**VA test**				**Total**	
		Fail*		**Pass**			
		***n***	**%**	***n***	**%**	***n***	**%**
Age	4 years	2	3.92	49	96.08	51	100.0
	5 years	7	4.29	156	95.71	163	100.0
	6 years	11	5.91	175	94.09	186	100.0
Total		20	5.00	380	95.00	400	100.0

*p = 0.732, chi-square test.

### Refractive errors

Refractive errors were the most prevalent cause of visual impairment (95%, 95% CI = 76.2, 98.8); astigmatism the commonest. Only one child had exotropia. Majority of the children were found to have compound myopic astigmatism (63.2%, 95% CI = 40.8, 80.9) (Table 5). Most of the children had refractive errors involving both eyes (*n* = 15). Of those who were found to have unilateral refractive error (*n* = 5), the left eye was more frequently affected then the right eye.

**Table 5:** Data of patients who had visual disorders

**Patient**	**Right eye**	**Refraction**	**Left eye**	**Refraction**	**Visual Impairment**
1	Compound myopic astigmatism	−1.0/-1.5/180	Compound myopic astigmatism	−2.25/-0.75/180	
2	Compound myopic astigmatism	−1.0/-2.75/180	Compound myopic astigmatism	−6.0/-3.25/180	PRESENT
3	Mixed astigmatism	0.25/-0.75/180	Mixed astigmatism	0.25/-0.5/180	
4	Compound myopic astigmatism	−1.5/-2.0/180	Compound myopic astigmatism	−1.5/-2.5/180	
5	Mixed astigmatism	0.75/-0.5/170	Mixed astigmatism	1.0/-1.25/180	
6	Simple myopia	−0.5	Simple myopia	−0.5	
7	Compound myopic astigmatism	−1.5/-0.5/019	Mixed astigmatism	2.0/-1.75/173	PRESENT
8	Compound myopic astigmatism	−2.75/-0.25/152	Compound myopic astigmatism	−2.25/-0.25/146	
9	Compound myopic astigmatism	−3.0/-0.25/180	Compound myopic astigmatism	−2.75/-0.25/180	
10	Compound myopic astigmatism	−3.0/-1.5/030	Compound myopic astigmatism	−1.25/-1.5/160	
11	Simple myopic astigmatism	0/-1.5/180	Simple myopic astigmatism	0/1.5/180	
12	Mixed astigmatism	0.75/-2.75/180	Mixed astigmatism	0.75/-2.25/180	
13	Compound myopic astigmatism	−2.5/0.75/180	Compound myopic astigmatism	−2.25/0.5/180	
14	Squint	PLANO	normal	PLANO	PRESENT
15	Compound myopic astigmatism	−3.0/-0.5/180	Compound myopic astigmatism	−2.5/0.5/180	
16	normal	PLANO	Compound myopic astigmatism	−2.25/-0.25/179	
17	normal	PLANO	Mixed astigmatism	0.5/-2.25/166	
18	normal	PLANO	Compound myopic astigmatism	−6.26/-1.5/009	PRESENT
19	normal	PLANO	Compound myopic astigmatism	−1.5/-0.5/163	
20	Compound myopic astigmatism	−5.0/-1.0/180	Compound myopic astigmatism	−1.5/1.0/180	PRESENT

## Discussion

The prevalence of visual impairment in our study was 5% (95% CI = 3.3%, 7.6%); mostly due to refractive errors. This figure is comparable with other populations. In the Baltimore Pediatric Eye Study, the percentage of preschool-aged children requiring spectacle correction was about 1.2% among White children and 1.8% among African-American children [3]. Jamali et al. reported that about 6.3% of the children entering school in Iran were at risk of amblyopia; mostly due to refractive errors [10]. In Hong Kong, about 4.4% of preschool children had either reduced visual acuity or strabismus [11]. Chia et al. reported a prevalence of 1.19% among Singaporean children aged 30 to 72 months [12]. While in Nepal, Karki reported that 5.97% of children aged 4 to 5 years have amblyopia [13].

The type of refractive error varies among different populations. Astigmatism was the commonest type of refractive error in our population. The axis of astigmatism in our study population was mostly with-the-rule. Significant refractive errors were uncommon. Hyperopia was found to be the most prevalent refractive error among preschool children in the Baltimore Pediatric Eye Study and in Iran [10,14]. In Singapore, where the prevalence of myopia is one of the highest in the world, preschool children had a high prevalence of myopia (11%-15%) [15,16]. The type of refractive errors in a population may change with time. Fan et al. found that in Hong Kong, the commonest type of refractive error shifted from astigmatism to myopia over a decade (1996–2007) [11].

Strabismus was found in one of the 20 children who did not pass the screening tests. The prevalent type of concomitant strabismus varies in different study population. Esotropia is more common in White population while exotropia in Asians [17]. In the Baltimore Pediatric Eye Study, the prevalence of strabismus was 0.3%; most due to esotropia [14]. Chia et al. reported a 0.80% prevalence of strabismus among preschool Singaporean Chinese. The exotropia-esotropia was about 7:1 [12].

The rate of testability increases with age [18]. As our sample consisted of older preschool children (aged 4 to 6 years), all 400 children in our study were testable for distant visual acuity and stereopsis. This is comparable with the findings of other studies [18,19]. This finding will provide a basis for future research to the evaluation of the effectiveness of these (Sheridan Gardiner Test Complete (Keeler, UK) and Langs stereotest) screening tests to detect amblyopia in our population.

### Limitation

The main limitation is due to the inherent weakness of a cross-sectional study. Data regarding the developmental milestone of the children were not collected. The presence of developmental delay may indicate the presence of associated visual impairment [20].

The non-respondent rate was about 11% and this may result in selection bias. The bias was influenced by caregiver characteristics where children who are already wearing spectacles may or may not be more prone to participate in the study. It is difficult to determine the direction of this bias. However, a higher respondent rate does not necessarily prevent selection bias [21].

The children were only tested for visual acuity and stereopsis. Comprehensive eye examinations were only offered to those who failed the screening tests. In other prevalence studies, all children were examined [3,10,12,13,16]. It is therefore difficult to compare our findings because of the statistics. Children with mild strabismus may not be detected as Hirschberg test and cover test was not performed on the field. Therefore strabismus is under-reported in this study. Retesting was not performed in this cross-sectional study. Although pre-test was given prior to actual screening, the possibility of poor cooperation cannot be ruled out.

The study was conducted among the urban population in Kuching district. It is reasonable to assume that the findings should apply to major towns with similar socioeconomic class such as Miri, Sibu or Bintulu. Generalization of the findings to the rural population should be viewed with caution. The prevalence of decreased presenting visual acuity may be higher in rural communities where spectacles are less available.

## Conclusion

The study is a small but important step in the effort to understand the problem of visual impairment among our preschool children. Our study showed that it is feasible to measure distant visual acuity and stereopsis in this age group. It provides some idea on the magnitude of the condition. However, the value of performing vision screening in preschool children is yet to be determined. Further research is needed in this aspect.

## Competing interests

The authors declare that they have no competing interests.

## Authors’ contributions

MP is the principal investigator. Data analysis was carried out by MP. All authors were involved with the drafting of the manuscript. All authors read and approved the final manuscript.

## Pre-publication history

The pre-publication history for this paper can be accessed here:

http://www.biomedcentral.com/1471-2415/13/16/prepub
